# Establishing an external quality assurance scheme for the detection of *Orientia tsutsugamushi* IgM in clinical samples: Strengthening quality control of scrub typhus diagnosis in Indian laboratories

**DOI:** 10.1371/journal.pntd.0014007

**Published:** 2026-02-27

**Authors:** Vimal Raj Ratchagadasse, Dinakaran Vasudevan, Ferdinamarie Sharmila Philomenadin, Haripriya Sivakumar, Nivedha Devanathan, Harmanmeet Kaur, Labanya Mukhopadhyay, Rahul Dhodapkar

**Affiliations:** 1 Regional Level Virus Research and Diagnostic Laboratory (RVRDL), Department of Microbiology, Jawaharlal Institute of Postgraduate Medical Education and Research (JIPMER), Puducherry, India; 2 Indian Council of Medical Research, V. Ramalingaswami Bhawan, Ansari Nagar, New Delhi, India; University of Liverpool, UNITED KINGDOM OF GREAT BRITAIN AND NORTHERN IRELAND

## Abstract

Scrub typhus, caused by *Orientia tsutsugamushi*, is a re-emerging zoonotic disease that is associated with significant morbidity and mortality in many parts of India. Early and accurate diagnosis, primarily through the detection of IgM antibodies, plays a pivotal role in patient management and disease surveillance. However, there is a paucity of diagnostic methods in our country, and only in recent years have more diagnostic assays been rolled out. To address the heterogeneity in the laboratory diagnosis of scrub typhus, an external quality assurance scheme (EQA Scheme) for the detection of O *tsutsugamushi* IgM (OTM) was conceptualized and implemented across a network of laboratories in India. This study provides detailed insight into the design, implementation, and outcomes of this EQA scheme initiative. Initially, a pilot phase was conducted among the 10 participant laboratories, and a total of 51 Virus Research and Diagnostic Laboratories (VRDLs) under DHR-ICMR undertaking OTM serology subsequently participated in the EQA Scheme. Proficiency testing (PT) panels consisting of 5 well-characterized and validated pooled serum samples were distributed to the participants for testing. Participating laboratories have employed various IgM detection methods, including ELISA and rapid immunochromatographic tests (ICTs). Performance was evaluated qualitatively based on the testing results and adherence to reporting timelines. Most laboratories demonstrated concordant results (n = 47) in OTM testing, while 4% of laboratories (n = 2) reported discordant results. The study underscores the vital role of an EQA scheme in ensuring quality-assured diagnostic testing for infectious diseases in the country and builds confidence in Indian laboratory results. Regular participation in such schemes, coupled with targeted capacity-building initiatives, can substantially improve laboratory diagnostics, facilitate timely clinical interventions, and contribute to a more robust national surveillance system. This model may serve as a foundation for strengthening quality assurance frameworks for other emerging infectious diseases in India and similar low- and middle-income settings.

## Author summary

Scrub typhus, caused by *Orientia tsutsugamushi*, is a major but underrecognized cause of acute febrile illness in many parts of India. Accurate laboratory diagnosis is critical for timely treatment and disease control. However, diagnostic performance can vary widely between laboratories due to differences in testing procedures, reagents, and quality control measures. To address this gap, we established an external quality assurance scheme (EQA Scheme) to assess and enhance the diagnostic capacity of Indian laboratories for detecting *O. tsutsugamushi*-specific IgM (OTM) antibodies. Our study involved the preparation and distribution of standardized, well-characterized serum panels to participating laboratories, followed by evaluation of their test results. We found considerable concordance in test performance across laboratories, highlighting the importance of standardized protocols and regular quality checks. This EQA Scheme initiative represents an important step toward improving the reliability and consistency of scrub typhus diagnosis in endemic regions. Strengthening diagnostic quality control will support better disease surveillance, patient care, and public health interventions in India and other tropical countries where scrub typhus remains a neglected threat.

## Introduction

Scrub typhus is a zoonotic infection caused by the bacterium *Orientia tsutsugamushi* and is transmitted by *Leptotrombidium* mites. Approximately one million cases of scrub typhus are reported annually throughout Asia, but this condition remains grossly underreported in India despite its significant public health burden [1,2]. The disease is fully treatable in its initial phase but causes significant morbidity and high mortality if not treated in a timely manner [3]. Scrub typhus is known to be endemic to the ‘tsutsugamushi triangle’ of the Asia-Pacific region, which covers South and Southeast Asia, North Australia, and the islands of the Indian and Pacific Oceans [1].

Scrub typhus is often underdiagnosed due to its nonspecific symptoms (fever, headache, body aches, with or without pathognomonic eschar at the mite bite location) and the country’s limited capacity for syndromic testing of infectious diseases. If left untreated, severe complications such as acute encephalitis, multiple organ failure, respiratory distress, and even death can occur. Despite its significant health impact, awareness of the disease has remained low among the general public and medical practitioners in many parts of India, thus contributing to its under recognition and misdiagnosis. This high incidence of scrub typhus, coupled with its often-severe outcomes, underscore the critical need for improved diagnostic measures, public awareness, and effective treatment protocols [4–6] to better identify and manage the disease and reduce its public health impact in endemic regions such as India.

Diagnostic laboratories must provide clinicians and patients with reliable and accurate scrub typhus test results for timely management of the disease and national disease surveillance efforts. Therefore, quality control becomes necessary for laboratory testing, with internal quality control (IQC) and external quality assessment (EQA) measuring intralaboratory and interlaboratory variability, respectively. There are a handful of Proficiency Testing (PT) panel providers in India; however, to the best of our knowledge, there has been no previous Indigenous EQA Scheme for laboratory testing of this priority pathogen. The regional importance of the disease and the prohibitive cost of internationally available panels were other driving factors for indigenous capacity building in PT panel preparation. The Indian Council of Medical Research (ICMR), in collaboration with the World Health Organization (WHO) and National Reference Laboratory (NRL) Australia, jointly initiated the development of serology and molecular EQA panels for infectious disease testing in the country in 2023. This study describes the launch of an EQA scheme for OTM detection across the DHR-ICMR VRDL network and reports the results of the first EQAS round.

## Methods

### Ethics statement

The study protocol was approved by the Institutional Ethics Committee (IEC No.: JIP/IEC/-OS/474/2023), permitting the use of leftover irreversibly deidentified serum samples with a waiver of consent in accordance with ICMR Guidance on ethical requirements for laboratory validation testing, February 2024 (**Available from:** https://main.icmr.nic.in/sites/default/files/upload_documents/Guidance_on_Ethical_Requirements_for_Laboratory_Validation_Testing.pdf).

### Conceptualization of the EQA scheme

The ICMR, WHO, and NRL Australia collaboratively launched a training program for the development of serology and molecular EQA panels between April and August 2023 for 17 ICMR-affiliated laboratories. The training was conducted in two phases: **Phase I** involved virtual sessions aimed at providing a foundational understanding of the EQA scheme, while **Phase II** consisted of hands-on training on PT panel preparation using various methodologies. Phase II training was held at the ICMR-National Institute of Virology (NIV), Pune, India. Following this, the ICMR rolled out EQA schemes across the selected laboratories for various priority infectious diseases under the project mode.

### Study site

The OTM serology EQAS was entrusted to DHR-ICMR Regional VRDL at the Jawaharlal Institute of Postgraduate Medical Education and Research (JIPMER).

### Preparation and Evaluation of Standard Operating Procedures (SOPs)

Standard operating procedures were prepared for various stages of EQA, as listed below:

- Planning for the EQA Scheme
- Sample bank preparation
- PT panel characterization
- Panel preparation
- Panel validation
- Panel distribution
- Data collection and analysis

### Planning for the EQA scheme

The list of laboratories for conducting the scrub typhus serological test was obtained from the ICMR VRDL portal. These laboratories were contacted to ascertain their interest in participating in the EQA programme and to gather information on the diagnostic kits they used, the latter for better characterization of the panel. The EQA scheme was designed as a biannual programme, with test panels distributed to participating laboratories to test the efficiency of testing OTM.

### Sample bank preparation and characterization

The sample bank was developed using irreversibly deidentified leftover samples archived at the RVRDL, JIPMER. These samples were tested and inventoried under appropriate storage conditions. Patient data were obtained from those who reported to the hospital with acute undifferentiated febrile illness and were referred to the laboratory for investigation. Leftover anonymized samples, including OTM reactive and OTM nonreactive samples that were nonreactive for antigen/antibody markers of the two most prevalent arboviral infections, Dengue and Chikungunya, were utilized for sample bank development. The samples were labeled according to the following sequence: Name of the source laboratory/year/Name of the Sample/R or NR (reactive or nonreactive)/Serial number of the samples. For example, JIPMER/2024/STM/R/01 refers to the first sample used for sample bank preparation; this sample was sourced from JIPMER in 2024, tested for OTM and found to be reactive. Reactive and nonreactive aliquots were stored at -80°C for further processing.

The EQA samples were characterized according to standard operating procedures. The sample aliquots were retrieved from the sample bank and retested using a reference test kit (Detect IgM ELISA; InBios International, Inc.). The scrub typhus Detect IgM ELISA System (InBios) and various scrub typhus IgM test kits commonly used in India, namely, J. Mitra & Co. Pvt. Ltd and Athense Dx Pvt. Ltd were used. Scrub typhus IgM Microlisa (J. Mitra) and Athense Dx Pvt. Ltd TRUSTwell scrub typhus IgM ELISA Kit (TRUSTwell) were used according to the manufacturer’s instructions. The InBios ELISA Kit was chosen as a reference test kit based on its common use by the participating laboratories. The proficiency testing laboratory confirmed that the participating laboratories routinely used one of the three aforementioned diagnostic kits for OTM testing by contacting them before the EQA programme to collect information on the diagnostic kits in use, as outlined earlier under the Planning for EQAS. All three kits were used for statistical analysis in this EQA exercise. In addition to Dengue and Chikungunya, blood-borne pathogens such as HIV, HBV, and HCV were also tested. Based on the results from the participating laboratories in the pilot study, the OD cutoff was set at 0.7 for the InBios kit, 0.4 for the J. Mitra kit, and 0.2 for the TRUSTwell kit. The assigned values for the PT items were determined based on the OD values reported by the participating laboratories during the pilot study. These assigned values represent the median OD values, also referred to as the consensus values derived from the participant results. Considering the assigned values, samples with similar OD values according to the InBios Kit were grouped and classified as follows: OD values between 0.700 and 1.500 were categorized as low reactivity, OD values between 1.500 and 2.000 were considered medium reactivity, and OD values of 2.000 or above were considered high reactivity. The categorized samples with similar absorbance values were pooled accordingly for PT panel preparation, with each pool comprising three individual samples with adequate sample volume. The minimum required sample volume for inclusion of a specimen in the serum pool was calculated as follows: the volume of samples required for the participant laboratories, the volume of samples required to rule out blood-borne viral pathogens, the volume of samples required for reference testing and various diagnostic kits employed by the participant laboratories for scrub typhus diagnosis using IgM ELISA. An additional volume was also considered for homogeneity and stability testing—10% of the samples were used for homogeneity testing, and 30% were used for stability testing.

We initially selected 63 reactive and 39 nonreactive samples. The reactive samples were classified into low, medium, and high reactive categories based on their OD values as described above. For each category, three reactive samples were pooled together to create a pool of 7 pools each of low, medium, and high reactive samples. Similarly, the nonreactive samples were grouped into sets of three to form a total of 13 nonreactive pools. These 13 pools were further divided into two groups: 7 pools were designated Non-Reactive Sample-01, and 6 pools were designated Non-Reactive Sample-02. Ultimately, five final pools were prepared—one each for the low-reactive, medium-reactive, high-reactive, nonreactive Sample-01, and nonreactive Sample-02 samples. The final pool contained approximately 5250 µl of sample.

Minimum sample volume required per individual sample in a panel or the pooling matrix

Samples for 51 participant laboratories: 50 µl X 51 = 2550 µl

Stability testing: 50 µl × 30 = 1500 µl

Samples for panel pool testing by test kits other than InBios: 50 µl × 4 = 200 µl

Additional 5% Samples: 50 µl × 6= 300 µl

10% Samples for Homogeneity Testing: 50 µl × 9 = 450 µl

The total volume of the approximate sample required for a single pool in the panel is 5000 µl. (The remaining sample in the pool was stored at -80°C until further use).

### Sample panel preparation and validation

The preparation of panels was performed using the reactive and nonreactive pools. The pooled sample vials were retested using a reference test and various test kits used by the participating laboratories to ensure repeatability and reproducibility. Pools that showed nonreactive results for blood-borne pathogens were utilized for panel preparation. Leakproof O-ring screw-capped cryotubes were appropriately labeled, and the nonreactive sample pools were aliquoted first, followed by the reactive sample pools, from low reactive to high reactive, to avoid cross-contamination. Good laboratory practices were followed to ensure this, processing one sample pool at a time. The pooled serum samples were evenly distributed in cryotubes, each containing 50 µl of sample, and then directly stored at -80°C until use.

To validate the integrity of the samples, the homogeneity and stability of the samples in the panel were assessed before shipment. Ten percent of the samples from each panel were randomly selected and thawed to test their integrity by detecting OTM antibodies. The homogeneity of the samples was tested twice within 24 hours. The stability of the samples was evaluated by testing 30% of the samples stored at different temperatures for different periods, ranging from freezing to ambient temperature (i.e., -80°C, -20°C, 4°C, 24°C (room temperature), 37°C and 42°C), for 144 hours, which mimics the approximate temperature and time taken to reach the participants’ regions. The stability period was determined based on the distance of the farthest laboratory (**Table 1**). The samples were found to be stable, as they had OD values similar to those of the original readings at different temperatures and time points. The temperature chosen for the shipment of the panels was -80°C Table 2,3.

**Table 1:** Table showing the required number of sample aliquots for stability testing across various temperature ranges and time intervals.

Temperature	Different time intervals					Total aliquot vials (No.)	Total volume of sample required ((µl)
	48 Hours	72 Hours	96 Hours	120 Hours	144 Hours		
**-80⁰C**	1	1	1	1	1	5	250
**-20⁰C**	1	1	1	1	1	5	250
**4⁰C**	1	1	1	1	1	5	250
**24⁰C**	1	1	1	1	1	5	250
**37⁰C**	1	1	1	1	1	5	250
**42⁰C**	1	1	1	1	1	5	250
**Total**	6	6	6	6	6	30	1500

**Table 2:** Homogeneity check of the samples according to the ISO 13528:2022 guidelines.

S. No.	Time Period	Standard Deviation of Proficiency Assessment (SDPA)	Absolute of difference between the Average Sample OD Values ABS ($\bar{x}$ -ȳ)	Stability check ABS ($\bar{x}$ -ȳ) ≤ 0.3 * SDPA	Result
1	Stability at 48 Hours	4.9	0.07	0.07 ≤ 1.47	Stable
2	Stability at 96 Hours	4.9	0.19	0.19 ≤ 1.47	Stable
3	Stability at 120 Hours	4.9	0.32	0.32 ≤ 1.47	Stable
4	Stability at 144 Hours	4.9	0.86	0.86 ≤ 1.47	Stable

**Table 3:** Stability check of the samples according to the ISO 13528:2022 guidelines.

S. No.	Standard Deviation of Proficiency Assessment (SDPA)	Between-samples Standard Deviation, Ss	Homogeneity check Ss ≤ 0.3 * SDPA	Result
1	4.9	1.4594	1.4594 ≤ 1.47	Homogeneous

### Sample Panel distribution to participating laboratories

Prior to dispatching the PT panels to all the designated laboratories, we conducted a pilot study with 10 laboratories in Puducherry and Tamil Nadu. The pilot study was performed to assess the feasibility of the EQA exercise, standardize the protocols for shipping the panels and instructing the participating laboratories and determining the cutoff value for the assay. The basic information about the Pilot study was given in Table 4A.

**Table 4.A:** Basic information about the laboratories that participated in the Pilot EQA Programme.

Number of Participants	10
**Geographical Distribution**	
Tamil Nadu	7
Puducherry	3
**Type of Laboratories**	
VRDLs (State Level)	2
VRDLs (Medical College Level)	5
*Medical College and Research Institutions*	
Government	8
Private	2
ELISA Kits Used	
InBios International, Inc.	8
J.Mitra & Co Pvt., Ltd., Results obtained from the 10 laboratories	2 Concordant

**Fig 1:**
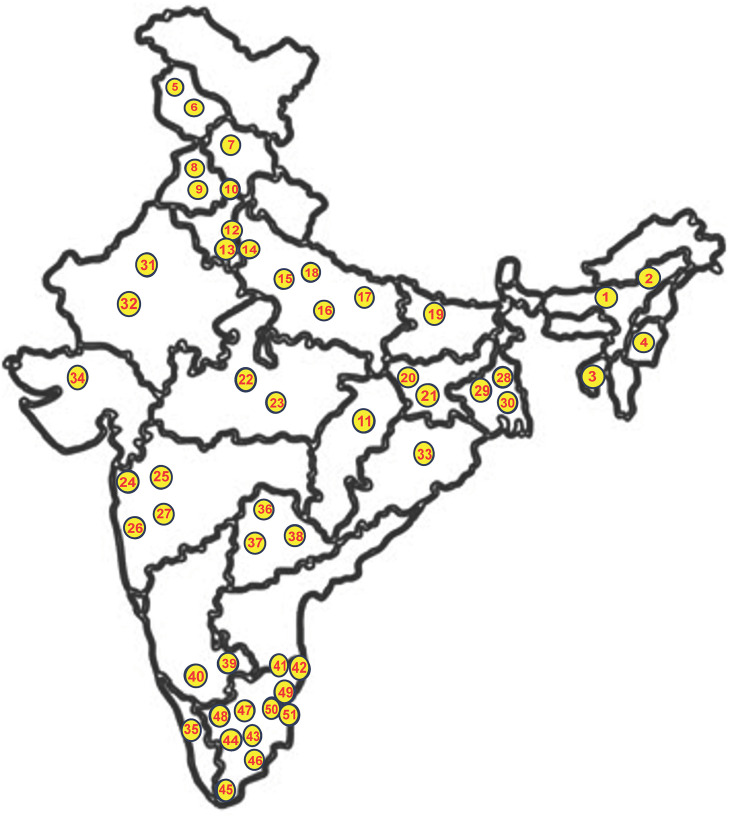
Geographic distribution of laboratories participating in the full EQA programme. We obtained the Map of India from the Survey of India portal, Department of Science and Technology (https://indiamaps.gov.in/). **Names of the laboratories that participated in the EQA Programme.** 1. Regional Medical Research Centre, Dibrugarh, Assam. 2. Gauhati Medical College, Guwahati, Assam. 3. Government Medical College, Agartala, Tripura. 4. Regional Institute of Medical Sciences (RIMS), Imphal, Manipur. 5. Govt Medical College, Srinagar, Jammu & Kashmir. 6. Sher-e-Kashmir Institute of Medical Sciences, Srinagar, Jammu & Kashmir. 7. Dr. Rajendra Prasad Government Medical College, Tanda, Himachal Pradesh. 8. Govt. Medical College, Amritsar, Punjab. 9. Govt. Medical College, Patiala, Punjab. 10. Government Medical College & Hospital, Chandigarh. 11. All India Institute of Medical Sciences (AIIMS) Raipur, Chhattisgarh. 12. Lady Hardinge Medical College, New Delhi. 13. Maulana Azad Medical college, New Delhi. 14. Dr. Ram Manohar Lohia Hospital and Atal Bihari Vajpayee Institute of Medical Sciences, New Delhi. 15. Government Institute of Medical Sciences, Noida, Uttar Pradesh. 16. King George’s Medical University (KGMU), Lucknow, Uttar Pradesh. 17. Dr. Gopal Nath, Banaras Hindu University (BHU), Varanasi, Uttar Pradesh. 18. Regional Medical Research Center, Gorakhpur, Uttar Pradesh. 19. Rajendra Memorial Research Institute of Medical Sciences (RMRIMS), Patna, Bihar. 20. MGM Medical College, Jamshedpur, Jharkhand. 21. Rajendra Institute of Medical Sciences (RIMS), Ranchi, Jharkhand. 22. All India Institute of Medical Sciences Bhopal, Madhya Pradesh. 23. National Institute of Research in Tribal Health (NIRTH), Jabalpur, Madhya Pradesh. 24. Kasturba Hospital for Infectious Disease, Mumbai, Maharashtra. 25. Seth **G.**S Medical college and KEM Hospital, Mumbai, Maharashtra. 26. Government Medical College (GMC), Aurangabad, Maharashtra. 27. Armed Forces Medical College, Pune, Maharashtra. 28. All India Institute of Medical Sciences (AIIMS) Kalyani, West Bengal. 29. ICMR - National Institute of Cholera and Enteric Diseases, Kolkata, West Bengal. 30. North Bengal Medical College, Darjeeling, West Bengal. 31. All India Institute of Medical Sciences (AIIMS) Jodhpur, Rajasthan. 32. Sawai Man Singh (SMS) Medical College, Jaipur, Rajasthan. 33. All India Institute of Medical Sciences Bhubaneswar, Odisha. 34. **B.**J. Medical College, Ahmedabad, Gujarat. 35. National Institute of Virology (NIV) Field Unit, Alapuzha, Kerala. 36. Osmania Medical College, Hyderabad, Telangana. 37. All India Institute of Medical Sciences, AIIMS Bibinagar (Hyderabad Metropolitan Region), Telangana. 38. Gandhi Medical College, Secunderabad, Telangana. 39. Mysore Medical College & Research Institute, Mysuru, Karnataka. 40. Bangalore Medical College & Research Institute, BMCRI, Bangalore, Karnataka. 41. ICMR- National Institute for Research in Tuberculosis, Chennai, Tamil Nadu. 42. The King Institute of Preventive Medicine and Research (KIPM&R), Chennai, Tamil Nadu. 43. Government Medical College, Theni, Tamil Nadu. 44. Madurai Medical College, Madurai, Tamil Nadu. 45. Tirunelveli Medical College, Tirunelveli, Tamil Nadu. 46. Government Thiruvarur Medical College, Thiruvarur, Tamil Nadu. 47. Govt. Mohan Kumaramangalam Medical College Hospital, Salem, Tamil Nadu. 48. Coimbatore Medical College, Coimbatore, Tamil Nadu. 49. Indira Gandhi Medical College & Research Institute (IGMCRI), Puducherry. 50. Pondicherry Institute of Medical Sciences (PIMS), Puducherry. 51. Sri Venkateshwara, Medical College (SVMCRH), Puducherry.

The full EQA panel was sent to 51 designated participating laboratories across the country. The EQA panel contained five frozen EQA samples transported at -80°C. The transportation agency ensured maintenance of the cold chain during transit and delivered the samples directly to the laboratories within 48 hours of shipment. Prior to delivery, participating laboratories were notified of the dispatch and expected delivery dates. Upon receipt, laboratories were required to promptly check the condition of the samples. The participating laboratories were instructed to store the samples at -80°C upon receipt or if immediate testing was not feasible. The geographic distribution and basic information of the laboratories that participated in the Full EQA programme are depicted in **Fig 1** and **Table 4B**. On the day of testing, the EQAS panel samples were processed for OTM serology as part of their routine diagnostic workflow following the instructions of the kit manufacturer. The participating laboratories were instructed to submit their test results within two calendar weeks of receiving the EQA. In addition to the test results, information was collected on the diagnostic kit used (including brand, lot/batch number, expiry date), details of the instruments and the calibration status of the equipment used, as well as the cutoff and OD values for each sample in the panel.

**Table 4.B:** Basic information about the laboratories that participated in the Full EQA Programme.

Number of Participants	51
**Type of Laboratories**	
Medical College and Research Institutions	
Government	49
Private	2
**ELISA Kits Used**	
InBios International, Inc.	28
J. Mitra & Co Pvt., Ltd. Trustwell ST IgM Kit Scrub Check Rapid Card	18 2 1
**Number of Participants**	51
**Type of Laboratories**	
Medical College and Research Institutions	
Government	49
Private	2
**ELISA Kits Used**	
InBios International, Inc.	28
J. Mitra & Co Pvt., Ltd.	18
Trustwell ST IgM Kit	2
Scrub Check Rapid Card	1

All laboratories with concordant results were graded as “A” for excellent performance and awarded a 100% performance score. The pass or fail status was determined based on whether the participant laboratories provided 100% concordant results or any discordant results (**Table 5**). Additionally, the type of diagnostic kits used by the participating laboratories was taken into account before assessing the concordance of their results.

**Table 5:** Performance grading based on qualitative analysis.

S. No.	Score	Score	Abbreviation
1	100%	Excellent	**A**
2	70 - 90%	Good	**B**
3	50 – 70%	Average	**C**
4	0 - 40%	Poor	**D**
5	DNP	Did not participate	**DNP**

### Data analysis

The EQA data were evaluated using several statistical parameters, including the mean absorbance value, assigned (median) value, standard deviation (SD), uncertainty, and percentage deviation or bias. The assigned value was defined as the median of all participant results after excluding outliers. Performance metrics such as the Q score and Z score were also calculated. The formulae used to compute uncertainty, percentage deviation or bias, the Q score, and the Z score are presented in **Table 6**. All graphical analyses were conducted using GraphPad Prism version 8.0.2 (California, USA). Additionally, a correlation analysis was conducted to evaluate the relationship between the type of instrument used (calibrated vs. noncalibrated) and the corresponding Z scores of the laboratories. The distribution patterns of absorbance values reported by participating laboratories for all five panel samples were analyzed using frequency distribution and represented through histogram plots in GraphPad Prism version 8.0.2. To further assess the distribution, the one-sample Kolmogorov‒Smirnov (KS) test was conducted using SPSS version 23 (Illinois, USA) to determine whether the absorbance values conformed to a specified distribution [7].

**Table 6:** The formulae used to compute the statistical parameters.

S. No.	Statistical Parameter	Formula	Reference
**1**	**Uncertainty**	Uncertainty = 1.25 x SD/√number of participants	[14]
**2**	**% Deviation/Bias**	Bias = (participant result ‒ assigned value)/(assigned value) × 100%.	[14]
**3**	**Q Score**	Q Score = (Reported Value – Assigned Value)/(Assigned Value)	[14]
**4**	**Z Score**	Z Score = (Reported Value – Assigned Value)/(SD)	[14]

## Results

### Sample panel validation

The integrity of the samples was validated by assessing the homogeneity and stability of the samples in the panel. The samples were confirmed to be homogeneous and stable based on the homogeneity and stability assessments conducted in accordance with the guidelines outlined in ISO 13528:2022 (**Table 2**).

### Results from the participating laboratories

The results obtained from all the laboratories in the pilot study were concordant (**Table 4** A). The detailed analyses of the OD values obtained from the participating laboratories in the Pilot study were given in **S1 File**.

Of the 51 laboratories enrolled in the full EQA scheme, 49 had final results before the submission deadline. The remaining two laboratories did not return with results. Analysis of the submitted data showed that 96% (47/49) of the laboratories reported concordant results. Two laboratories did not qualify for the EQA exercise, scoring 60% (Out of the results from the 5 samples, 3 were correct and 2 were wrong results) and 80% (Out of the results from the 5 samples, 4 were correct and 1 was wrong result), respectively. Among these two laboratories with discordant results, one received a “B” grade, and the other a “C” grade. Results from laboratories are graded A (100%) to D (0–40% concordance) as per Table 5.

**Fig 2:**
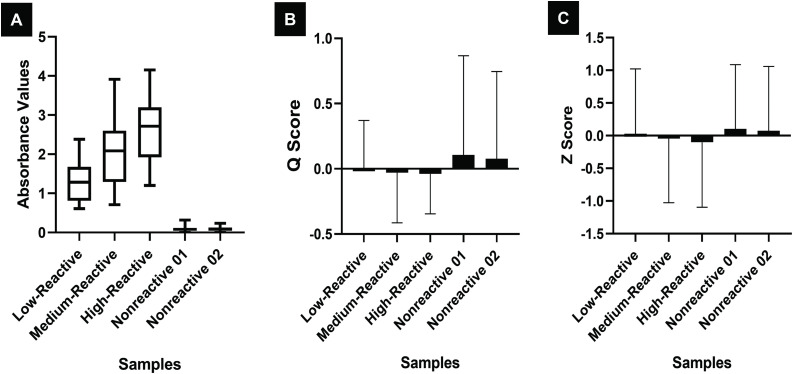
Absorbance values (A) and performance scores—the Q score (B) and Z score(C)—of samples tested by participant laboratories using the InBios ELISA Kit.

**Fig 3:**
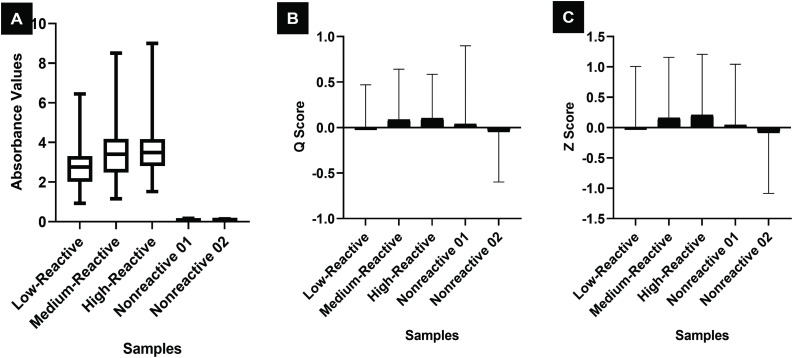
Absorbance values (A) and performance scores—the Q score (B) and Z score (C)—of samples tested by participant laboratories using the J. Mitra ELISA Kit.

Among the 49 laboratories that submitted their data, two laboratories reported discordant results and one laboratory used a Rapid Immunochromatographic Card Test (ICT) test instead of an ELISA, resulting in 46 eligible laboratories for analysis. Of these 46 laboratories, 27 used the InBios ELISA kit, 17 used the J. Mitra ELISA kit, and 2 used the Trustwell ELISA kit. Because only two laboratories used the Trustwell kit, their data were excluded from further analysis. Thus, the final dataset for detailed evaluation included 44 laboratories—27 using the InBios ELISA kit and 17 using the J. Mitra ELISA kit. The detailed statistical analysis results for the laboratories using the InBios ELISA Kit and the J. Mitra ELISA Kit were provided in Tables 7 and 8, respectively. The absorbance values (OD) of each sample in the panel, as determined using the InBios and J. Mitra ELISA Kits, were collected from the participating laboratories and are illustrated using box plots in **Figs 2 and 3**. The corresponding Q score and Z score are represented in the form of histograms in **Figs 2 and 3**.

**Table 7:** Statistical analyses of the results reported from laboratories using an InBios ELISA kit.

S. No.	Analysis	Samples				
		Low Reactive	Medium Reactive	High Reactive	Non-Reactive 01	Non-Reactive 02
**1**	**Mean**	1.288	2.022	2.609	0.091	0.094
**2**	**Median/Assigned Value**	1.285	2.084	2.715	0.082	0.087
**3**	**Standard Deviation**	0.472	0.802	0.832	0.062	0.058
**4**	**Uncertainty**	0.11	0.19	0.20	0.02	0.01
**5**	**Q Score**	0.00	-0.03	-0.04	0.11	0.08
**6**	**Z Score**	0.03	-0.04	-0.10	0.10	0.08
**7**	**% Deviation/Bias**	0.27	-2.96	-3.91	10.66	7.63

**Table 8:** Statistical analyses of the laboratory results were performed using a J. Mitra ELISA Kit.

S. No.	Analysis	Samples				
		Low Reactive	Medium Reactive	High Reactive	Non-Reactive 01	Non-Reactive 02
**1**	**Mean**	2.769	3.706	3.841	0.056	0.062
**2**	**Median/Assigned Value**	2.76	3.4072	3.488	0.054	0.065
**3**	**Standard Deviation**	1.292	1.887	1.690	0.046	0.036
**4**	**Uncertainty**	0.39	0.57	0.51	0.01	0.01
**5**	**Q Score**	0.00	0.09	0.10	0.04	-0.05
**6**	**Z Score**	0.01	0.16	0.21	0.04	-0.08
**7**	**% Deviation/Bias**	0.31	8.78	10.12	3.80	-4.66

### Data analysis of the full EQA programme

The performance scores, namely, the Q score and Z score, ranged from -0.5 to +0.5 for the majority of samples, with a few outliers observed (Figs 2 and 3). Among the laboratories that used the InBios ELISA Kit, three reported questionable Z scores, and one laboratory had an unacceptable Z score. Similarly, among those using the J. Mitra ELISA Kit, one laboratory showed a questionable Z score, and the other had an unacceptable Z score. A Z-score between –2 and +2 is considered acceptable. Z-scores from –3 to –2 or from +2 to +3 are regarded as questionable, while Z-scores below –3 or above +3 are deemed unacceptable. Accordingly, four laboratories showed questionable Z-scores and two laboratories showed unacceptable Z-scores. Except for one laboratory in each group, for which an EQAS score of 80 was recorded, all the other laboratories achieved an EQAS score of 100. The correlation analysis conducted to evaluate the relationship between the type of instrument used (calibrated vs. noncalibrated) and the corresponding Z scores of the laboratories revealed a weak correlation, with correlation coefficients of 0.2 for laboratories using the InBios ELISA Kit and 0.3 for those using the J. Mitra ELISA Kit (Table 9).

**Table 9:** Correlations between laboratory data measured by calibrated instruments and corresponding Z scores.

S. No.	Name of the Instrument	Number of labs that turned up with Results	Number of labs using calibrated instrument	Number of Labs using noncalibrated instrument	Correlation between labs that used Calibrated Instruments and their corresponding Z Score
**1**	**ELISA Reader**	49	29	20	0.2 to 0.3
**2**	**ELISA Washer**	49	28	21	
**3**	**Pipettes (Both Single Channel and Multi-Channel)**	49	39	10	

The p values for all the samples tested using the one-sample Kolmogorov‒Smirnov (KS) test were found to be greater than the significance threshold of 0.05, indicating that the absorbance values for each sample followed a normal distribution **(Tables A and B in** S2 File).

## Discussion

The EQA Scheme is a program designed to evaluate and enhance the quality of laboratory testing by comparing a laboratory’s results with those of other laboratories. This process ensures accurate and reliable reporting. EQA Scheme programs provide an objective assessment of laboratory performance, identifying areas for improvement. Laboratories participate by testing samples provided by an external organization, and their results are analyzed in comparison to those of other participants. This comparison helps detect and correct errors or biases in testing procedures, supporting the development of strong quality management systems.

Participation in EQA programs is often essential for laboratory accreditation and recognition. These programs enable laboratories to benchmark their performance at the national and international levels while fostering continuous improvements in testing processes. EQA schemes are classified into two types: (A) **proficiency testing (PT)** – Laboratories analyze samples with undisclosed results and receive feedback on their performance. (B) **Rechecking or Retesting** – Samples are sent to higher-level or peer laboratories for verification through retesting.

Previous Indian studies have consistently shown that OTM ELISA has substantially higher diagnostic sensitivity and specificity than the Weil–Felix test, which is prone to cross-reactivity [1,8]. The EQA Scheme OTM ELISA (Serology) is a simultaneous and continuous scheme. To our knowledge, this is the first study from India on the EQA Scheme to assess the use of serological testing for the detection of OTM by ELISA. The participating laboratories are scored based on their results. The results will be kept confidential between the participants and the provider. The results can be revealed to a regulatory body with written consent from the participant. However, in exceptional circumstances, the results from the participant will be provided to the regulatory body, and the participant will be notified of this action in writing.

The current study yielded 96% concordant results from the laboratories across all analyses. Two out of the 49 participating laboratories reported discordant findings, and one laboratory used a Rapid ICT card to test the EQA panel instead of an ELISA-based method. Among the 46 laboratories included in the comparative evaluation, clear performance differences were observed between those using the InBios ELISA kit and those using the J. Mitra ELISA kit. Laboratories employing the InBios kit demonstrated lower mean absorbance values, lower median assigned absorbance values, and correspondingly lower measurement uncertainties. These findings suggest that the InBios kit may offer more consistent assay performance and reduced variability across laboratories.

In terms of quality assessment, 44 of the 46 laboratories achieved acceptable Z-scores (within the –2 to +2 range), indicating generally reliable inter-laboratory performance. However, two laboratories reported unacceptable Z-scores (below –3 or above +3), highlighting potential issues in assay execution, calibration practices, or kit handling. A similar pattern was observed in the EQAS assessment, where 44 laboratories achieved a perfect score of 100, while two laboratories scored 80, further reinforcing the variability seen in a small subset of participants. Interestingly, only a weak correlation (r = 0.2–0.3) was observed between instrument calibration status and absorbance values. This suggests that while calibration quality may contribute to some variability, it is unlikely to be the sole factor influencing inter-laboratory differences. Other elements, such as kit lot variation, operator expertise, adherence to assay protocols, and environmental conditions, may play a more significant role and warrant further investigation. Additionally, laboratories with unacceptable or questionable Z scores showed greater percentage deviation (%D) or bias.

An EQA study for the serological diagnosis of scrub typhus was previously conducted in China, where the S3 antigen of *Orientia tsutsugamushi* was detected using serological methods. Out of nine participating laboratories, eight reported concordant results for *O. tsutsugamushi* [9]. In India, EQA studies have focused primarily on the molecular diagnosis of COVID-19 and respiratory viruses such as influenza by comparing Ct values and corresponding viral copy numbers in patient throat swab samples [10–12]. However, serological EQA studies have also been conducted in India for dengue, chikungunya, and Japanese encephalitis virus infections. In that study, three separate panels, each comprising six human serum samples (two Reactive and four Nonreactive) for anti-dengue, anti-chikungunya, and anti-Japanese encephalitis virus IgM antibodies, were distributed to 124 laboratories across India during 2018–19 and 2019–20 to assess the sensitivity, specificity, and reproducibility of serological testing. The average concordance across all three viruses was 98% in both years [13].

The major limitation of this study is that only OD values and not E-ratios were used for downstream statistical analysis. E-Ratios were not used for the statistical analysis because the cutoff values used to determine E-Ratios vary for each and every laboratory based on their geographic location and the diagnostic kit used for the study. The present study on the scrub typhus EQA Scheme included 51 participant laboratories across the country. The study was well planned and implemented following a successful pilot EQA involving 10 laboratories. Additionally, we did not standardize this EQA for the Weil Felix test. The results and analyses of this EQA Scheme for scrub typhus should be further validated by including a larger number of laboratories (100–200) in subsequent years. The EQA Scheme will continue to be implemented on a broader scale across the country in the future.

## Conclusion

The EQA Scheme for the detection of OTM was successful, as evidenced by the results obtained from the participant laboratories. This study was also conducted with a comparatively small number of participating laboratories. To determine the efficacy of the test performance of laboratories across the country, such a study must be implemented on a larger scale with the participation of a larger number of laboratories. Furthermore, this EQA scheme for the detection of OTM needs to be performed continuously to assess the quality of testing laboratories in India.

## Supporting information

S1 File Analyses of the Absorbance values obtained from the participating laboratories in the Pilot EQA study. (XLS)

S2 File Table A. Results of the one-sample Kolmogorov–Smirnov (KS) test for the samples tested by participant laboratories using the InBios ELISA Kit. Table B. Results of the one-sample Kolmogorov–Smirnov (KS) test for the samples tested by the participant laboratories using the J. Mitra ELISA Kit. (DOC)

S3 File Absorbance values obtained from the participating laboratories in the Full EQA study. (XLS)
