# Correction: Establishing an external quality assurance scheme for the detection of *Orientia tsutsugamushi* IgM in clinical samples: Strengthening quality control of scrub typhus diagnosis in Indian laboratories

**DOI:** 10.1371/journal.pntd.0014324

**Published:** 2026-05-18

**Authors:** Vimal Raj Ratchagadasse, Dinakaran Vasudevan, Ferdinamarie Sharmila Philomenadin, Haripriya Sivakumar, Nivedha Devanathan, Harmanmeet Kaur, Labanya Mukhopadhyay, Rahul Dhodapkar

## Notice of Republication

This article was republished on April 17, 2026, to address an issue identified post-publication. Please download this article again to view the correct version.
