# Supplementary material for: Establishing an external quality assurance scheme for the detection of Orientia tsutsugamushi IgM in clinical samples: Strengthening quality control of scrub typhus diagnosis in Indian laboratories
Source: PLoS Negl Trop Dis. 2026 Feb 27;20(2):e0014007. doi: 10.1371/journal.pntd.0014007 (PMC12948066; doi:10.1371/journal.pntd.0014007)
Supplement: S2 File — (DOC) [file pntd.0014007.s002.doc]

**S2 File**

**Table A. Results of the one-sample Kolmogorov‒Smirnov (KS) test for the samples tested by participant laboratories using the InBios ELISA Kit**

| **Contents** | **Low Positive**  **Samples** | **Medium Positive**  **Samples** | **High Positive** | **Non-Reactive Samples-01** | **Non-Reactive Samples-02** |
| --- | --- | --- | --- | --- | --- |
| **Number of Samples N** | 27 | 27 | 27 | 27 | 27 |
| **Normal Parameters^a,b^ Mean** | 1.2885 | 2.0223 | 2.6088 | 0.0907 | 0.0936 |
| **Std. Deviation** | 0.47214 | 0.80238 | 0.83248 | 0.06238 | 0.05824 |
| **Most Extreme Differences Absolute** | 0.104 | 0.105 | 0.125 | 0.177 | 0.134 |
| **Positive** | 0.104 | 0.105 | 0.125 | 0.177 | 0.134 |
| **Negative** | -0.075 | -0.093 | -0.116 | -0.136 | -0.063 |
| **Test Statistic** | 0.104 | 0.105 | 0.125 | 0.177 | 0.134 |
| **Asymp. Sig. (2-tailed)** | 0.200^c, d^ | 0.200^c,d^ | 0.200^c,d^ | 0.029^c^ | 0.200^c,d^ |

**a. The test distribution is normal. b. Calculated from the data. c. Lilliefors Significance Correction. d. This is the lower bound of the true significance.**

**Table B. Results of the one-sample Kolmogorov‒Smirnov (KS) test for the samples tested by the participant laboratories using the J. Mitra ELISA Kit**

| **Contents** | **Low Positive**  **Samples** | **Medium Positive**  **Samples** | **High Positive** | **Non-Reactive Samples-01** | **Non-Reactive Samples-01** |
| --- | --- | --- | --- | --- | --- |
| **Number of Samples N** | 17 | 17 | 17 | 17 | 17 |
| **Normal Parameters^a,b^ Mean** | 2.7687 | 3.7064 | 3.8409 | 0.0561 | 0.0620 |
| **Std. Deviation** | 1.29164 | 1.88684 | 1.6901 | 0.04644 | 0.03588 |
| **Most Extreme Differences Absolute** | 0.177 | 0.232 | 0.227 | 0.198 | 0.140 |
| **Positive** | 0.177 | 0.232 | 0.227 | 0.198 | 0.134 |
| **Negative** | -0.103 | -0.154 | -0.154 | -0.114 | -0.140 |
| **Test Statistic** | 0.177 | 0.232 | 0.227 | 0.198 | 0.140 |
| **Asymp. Sig. (2-tailed)** | 0.163^c^ | 0.016^c^ | 0.020^c^ | 0.076^c^ | 0.200^c,d^ |

**a. The test distribution is normal. b. Calculated from the data. c. Lilliefors Significance Correction. d. This is the lower bound of the true significance.**
